# Serum metallome in pregnant women and the relationship with congenital malformations of the central nervous system: a case-control study

**DOI:** 10.1186/s12884-019-2636-5

**Published:** 2019-12-05

**Authors:** Jacopo Troisi, Luigi Giugliano, Laura Sarno, Annamaria Landolfi, Sean Richards, Steven Symes, Angelo Colucci, Giuseppe Maruotti, David Adair, Marco Guida, Pasquale Martinelli, Maurizio Guida

**Affiliations:** 1 Department of Medicine and Surgery, Scuola Medica Salernitana, University of Salerno, Salerno, Italy; 2 THEOREO Srl Spin-off Company of the University of Salerno, Salerno, Italy; 3 Department of Neurosciences, Dentistry and Reproductive Sciences, University of Naples “Federico II”, Naples, Italy; 4 Department of Biology, Geology and Environmental Sciences, University of Tennessee at Chattanooga, 615 McCallie Ave, Chattanooga, TN 37403 USA; 5 Department of Chemistry and Physics, University of Tennessee at Chattanooga, 615 McCallie Ave, Chattanooga, TN 37403 USA; 6 Department of Obstetrics and Gynecology, University of Tennessee College of Medicine, Chattanooga, TN USA; 7 Department of Biology, University of Naples “Federico II”, Naples, Italy

**Keywords:** Aluminum, Congenital malformations, Central nervous system, Metals

## Abstract

**Background:**

Congenital malformations of the central nervous system (CNS) consist of a wide range of birth defects of multifactorial origin.

**Methods:**

Concentrations of 44 metals were determined by Inductively Coupled Plasma Mass Spectrometry in serum of 111 mothers in the second trimester of pregnancy who carried a malformed fetus and compared them with serum concentrations of the same metals in 90 mothers with a normally developed fetus at the same week of pregnancy.

Data are reported as means ± standard deviations.

**Results:**

We found a direct relationship between congenital defects of the CNS and maternal serum concentration of aluminum: it was statistically higher in women carrying a fetus with this class of malformation, compared both to mothers carrying a fetus with another class of malformation (6.45 ± 15.15 μg/L Vs 1.44 ± 4.21 μg/L, *p* < 0.0006) and to Controls (i.e. mothers carrying a normally-developed fetus) (6.45 ± 15.15 μg/L Vs 0.11 ± 0.51 μg/L, p < 0.0006). Moreover, Aluminum abundances were below the limit of detection in the majority of control samples.

**Conclusion:**

CAluminum may play a role in the onset of central nervous system malformations, although the exact Aluminum species and related specific type of malformation needs further elucidation.

## Background

Congenital malformations of the central nervous system (CNS) represent a wide range of congenital birth defects, for which the prevalence is about 2.3 per 1000 births in Europe [1] *.* The prevalence is likely even higher if we also consider pregnancies complicated by CNS malformations that end in spontaneous abortions or elective termination of pregnancy [2] .

CNS malformations can be classified according to the phase of embryological development in which they occur: thus, we distinguish among Neural Tube Defects (NTDs), that are the most common; Disorders of Regionalization, Disorders of Cortical Development, and Disorders of Myelination.

The etiologies of CNS malformations can be extremely different; sometimes they can occur in the context of a monogenic or chromosomal disorder or they can result from known teratogenic agents, such as infections or certain drugs. However, most CNS malformations do not occur in the context of a well-known causative agent [3] and it is generally accepted that the majority of CNS malformations have multifactorial origins.

It has been reported that exposure to metals can lead to neurotoxicity. Indeed, the nervous system has specific characteristics that make it highly susceptible to metals, due to its complex structure and long period of development. Metals can cross the blood-brain barrier and accumulate in cerebrospinal fluid [4]. Moreover metals can alter enzymes involved in ATP production, being particularly dangerous in very metabolically active tissues, like the brain [5].

Exposure to lead can result in increased permeability of the blood-brain barrier, brain swelling, herniation, ventricular compression, hemorrhages, thrombosis and arteriosclerosis [6].

There is evidence that Aluminum (Al) and Manganese (Mn) correlate with neurodegenerative disorders (Alzheimer Disease and Parkinsonism, respectively) [7]. Moreover, Al exposure has been reported to be associated with balance disorders, memory and concentration defects, fatigue, irritability and depression [4]. Al alters cholinergic neurotransmission, induces oxidative stress and causes iron accumulation [8–10] .

Exposure to heavy metals has been correlated with the occurrence of NTDs in multiple studies. Liu et al. [11] found that placental concentration of manganese was higher in cases having a fetus affected by NTDs than in controls. Another study conducted in a Chinese population [12], found that higher placental concentrations of mercury was associated with an elevated risk of NTDs in newborns. Ramírez-Altamirano et al. [13] found an increase of Al and silver in the hair of newborns with neural tube pathologies. Gatti et al. [14] found Al contamination in the liver and kidney of fetuses with NTDs and none in fetuses without such a complication. Huang et al. [15] found a correlation between the prevalence of NTDs and the amount of tin, lead, nickel, iron, copper and Al in the soil of numerous Chinese sites. Cengiz et al. [16] found serum copper and whole-blood lead (Pb) levels were significantly higher in women whose pregnancies were terminated as a result of diagnosis of NTDs, when compared to controls. The present work is an attempt to relate metal exposure to the development of CNS malformations. In particular, the aim of this study was to investigate the relationship between specific congenital defects and maternal exposure to heavy metals, quantified by a whole metallome, defined as a comprehensive quantitation of metals and metalloids in maternal serum.

## Methods

### Population, study design and setting

This was a case-control study conducted in three hospitals, University of Naples “Federico II”, University of Salerno and Hospital “G. Moscati” of Avellino, in Campania, Southern Italy, from January 2011 to December 2013.

Pregnant women with a diagnosis of fetal malformations or fetal chromosomal abnormalities (Cases) were compared with healthy controls (CTRLs).

All Cases were recruited at the time of second trimester termination of pregnancy, while CTRLs delivered normally developed fetuses, and were recruited during their second trimester routine anomaly scan.

The presence or absence of fetal malformations or chromosomal abnormalities was defined based on ultrasound examination or karyotype and confirmed by postmortem autopsy by an expert pathologist or after pediatric examination of the newborns.

Exclusion criteria for Cases were: maternal age > 40 years old, twin pregnancy, pregnant women committed to carrying the pregnancy to term, TORCH (Toxoplasma, Rosolia, Citomegalovirus, Herpes) complex infection, or CNS defects with a known genetic cause.

The study was approved by the local ethics committee (IRB n.4/2013). A written consent form was signed by each participant at the time of enrollment.

Enrolled patients completed a questionnaire addressing clinical history and demographic characteristics and a complete obstetric visit was performed at enrollment to collect a thorough medical history. The investigations determined the presence of any known etiological factors of malformations, including: history of infections; malnutrition or metabolic disease (e.g. diabetes); drug use (e.g., thalidomide, anticoagulants, chemotherapeutic agents) and drug addiction (e.g., cannabis, cocaine, heroin); radiological investigations (e.g. X-rays, Computerized Tomography); and/or history of genetic syndromes.

Cases were subsequently divided into two groups:

- CNS group which included all CNS malformations with unknown etiology;
- Other group, which included all other malformations or chromosomal abnormalities.

### Samples collection

Human tissue collection strictly adhered to the guidelines outlined in the Declaration of Helsinki IV edition. All blood was collected using a BD Vacutainer (Becton Dickinson, Oxfordshire, UK) blood collection red tube (no additives). Blood samples of the Cases were collected in the second trimester immediately before termination of pregnancy and before any drug administration. Blood samples of CTRLs were collected during the second trimester routine anomaly scan. After centrifugation, serum was transferred to cryovials and immediately frozen to − 80 °C until the time of analysis. All patients were asked to respect a 12-h fast before blood collection.

### Metals concentration

Serum samples were analyzed with an ICP-QMS Bruker 820-MS (Bruker Daltonics, Billerica, MA). Operational parameters were: Plasma flow: 18 L/min, Auxiliary flow: 1.8 L/min, Sheath Gas: 0.14 L/min, Nebulizer flow: 0.98 L/min, RF power: 1.40 kW, Pump rate: 4 rpm, Stabilization delay: 20 s, First Extraction Lens: − 40 V, Second Extraction Lens: − 166 V, Third Extraction Lens: − 234 V, Corner Lens: − 208 V, Mirror Lens left: 29 V, Mirror Lens right: 26 V, Mirror Lens bottom: 30 V; CRI parameters: Skimmer Gas: H_2_ at 50 ml/min, Sample Gas: He at 10 ml/min; dwell time, 50,000 μs; no. of scan replicate: 10, no. replicate for sample: 5. High purity He (99.9999% He, AirLiquide Srl, Italy) and H_2_ (99.9999% H_2_, produced by the DBS H_2_ generator PGH2–300) were used, in order to minimize the potential problems caused by unidentified reactive contaminant species in the cell. The high radio frequency power (1400 W) helped maintain plasma stability. All chemicals were of the highest purity grade that is commercially available. Before use, all glassware and plastic containers were cleaned by washing with 10% ultra-pure grade HNO_3_ for at least 24 h, and then rinsed copiously with 18 MΩ water (produced by the Direct-Q-UV, Millipore, Billerica, MA, USA) before use. Peltier cooled ICP spray chamber temperature was setted at 3 °C. This ensure temperature stability and to reduce the water vapor production in the nebulizer gas flow. Standard solutions and samples were analyzed by means the SPS3 autosampler (Varian Inc., Mulgrave, Australia) coupled to the ICP mass spectrometer After collection, all serum samples were anonymized (a 3-letter and 3-number code was assigned) and stored at − 80 °C until analysis. Serum samples were pre-heated before the analysis keeping them at room temperature for two hours before sample preparation. After a gently vortex mixing (30 s at 300 rpm), 500 μL was diluted with 100 μL 0.1% (V/V) Triton-X-100 solution (Sigma-Aldrich, Seelze, Germany). and filled up to 5 mL with a 0.5% (v/v) NH_4_OH (Merck, Darmstadt, Germany) in a 10 mL polypropylene tube using a 5 mL bottle-top dispenser (Brand, Wertheim, Germany). Samples were then homogenized with an oribital stirrer KS3000i (IKA, Staufen, Germany).

Standard addition method was used for calibration according to Heitland and Köster [17]. Briefly, five hundred microliters of the serum sample were diluted up to 5 mL. A stock standard solution containing the metals under investigation were prepared in 100 mL polypropylene flasks diluting 20 mg/L multi-element standard solutions for Inductively Coupled Plasma-Mass Spectrometry (ICP-MS) (Ultrascientific, North Kingstown, USA). These solutions were at defined volumes to a mixture composed of of 500 μL serum, 100 μL 0.1% (v/v) Triton-X-100 solution in 10 mL plastic (polypropylene) tubes for autosampler (Merck, Darmstadt, Germany). These solutions were finally filled up to 5 mL using 0.5% NH_4_OH. Dilutions were carried out with variable volume pipettes (volumes 50–1000 μL) and a bottle dispenser with adjustable volumes from 1 to 5 mL (Eppendorf, Hamburg, Germany). The isotopes analyzed were: ^7^Li, ^9^Be,^27^Al, ^49^Ti, ^51^V, ^52^Cr, ^55^Mn,^59^Co, ^65^Cu, ^66^Zn, ^71^Ga, ^78^Se, ^85^Rb, ^88^Sr, ^90^Zr, ^93^Nb, ^98^Mo, ^101^Ru, ^107^Ag, ^114^Cd, ^115^In, ^121^Sb ^125^Te, ^137^Ba, ^140^Ce, ^141^Pr, ^146^Nd, ^147^Sm, ^153^Eu, ^157^Gd, ^163^Dy, ^166^Er, ^169^Tm, ^172^Yb, ^178^Hf, ^181^Ta, ^182^W, ^185^Re, ^189^Os, ^193^Ir, ^195^Pt, ^202^Hg, ^205^Tl, ^206,207,208^Pb. These 15: ^9^Be, ^27^Al, ^51^V, ^52^Cr, ^55^Mn,^59^Co, ^65^Cu, ^66^Zn, ^78^Se, ^107^Ag, ^114^Cd, ^121^Sb,^202^Hg, ^205^Tl, ^206,207,208^Pb were quantified by calibration curve while the others were quantified by semi-quantitative method which uses an average response factor based on neighboring elements on the periodic table. This approach can have the same accuracy as calibration with individual standards [18]. Samples were analyzed in a random computer-generated sequence. Another multi-element calibration solution of a different production lot from the same manufacturer was used to verify the elemental concentrations in all multi-element calibration solutions. All solutions were analyzed by aspirating (with a Y–connection) an internal standard solution of 10 μg/L of ^6^Li, ^45^Sc, ^72^Ge, ^89^Y, ^103^Rh ^159^Tb, ^165^Ho, ^209^Bi in 2% (v/v) HNO_3_ (Ultrascientific, North Kingstown, USA). Internal quality assurance was obtained analyzing the Clinchek® Whole Blood Control Level 1–3 (Recipe, Munich, Germany) and Seronorm® Trace Elements Whole Blood Control Level 1–3 (Sero AS, Billingstad, Norway).. Repeat analysis of method blanks showed that all materials and reagents were free of metal contamination. The limit of detection (LOD) for each element was evaluated with two methods: first, as the concentration that corresponds to a signal equal to the average blank signal plus 3 times the standard deviation of the signal from 10 replicates of a blank sample (LOD = Avg_blank_ + 3σ_blank_); second as 3 times the concentration relative to the signal from the standard deviation of the y-intercept of the calibration curve (s_x/y_) [19]. The higher value was used as LOD. All analyses were replicated three times and mean values calculated to be used for statistical testing.

### Statistical analysis

Study data were collected and managed using REDCap electronic data capture tools hosted at the INFN Institute of the University of Salerno (Italy) [20]. Statistical analysis was performed using Statistica software (StatSoft, Oklahoma, USA) and Minitab (Minitab Inc., Pennsylvania, USA).

The normal distribution of all data was verified using the Kolmogorov-Smirnov test. Logarithmic transformation was performed on skewed variables. For descriptive purposes, continuous variables were reported as mean ± standard deviation (SD) as untransformed values, while categorical variables were reported as number (percentage). Independent two-tailed t-tests were used to compare parameters between two groups. A ɑ-value< 0.05 was considered statistically significant. The ɑ-value was adjusted according to Bonferroni setting to 0.05/88 = 0.0006. Values for concentrations below the limit of detection (LOD) were imputed as LOD divided by square root of 2 [21]. For parameters of more than one category, Analysis of Variance (ANOVA) was performed. Significant differences were followed by a Tukey’s post-hoc.

### Multivariate statistics analysis

Two classification models were used to verify if metal distribution was globally different among the CTRLs, Cases (CNS malformations) and Other classes. One model uses Principal Component Analysis (PCA), an unsupervised algorithm finding the directions that best explain variance in a dataset (X) without referring to class labels (Y). The dataset, comprised of all elemental concentrations of all samples, was median-centered and log transformed. The data were then summarized into many fewer variables (called principal components) which are weighted averages of the original variables (where the weighting profiles are called loadings). The PCA analysis was performed using the *prcomp* package for R Software (Foundation for Statistical Computing, Wien, Austria). Calculations were based on singular value decomposition. A separate model used to determine if metal distributions are globally different between the Control, Case and Other classes was Partial Least Squares-Discriminant Analysis (PLS-DA), which is a supervised method that uses multivariate regression techniques to extract, via linear combination of original variables (X, the metal concentrations), the information that can predict class membership (Y, CNS malformation or Control). We used the R pls package to perform the PLS regression, using the plsr function [22].. The wrapper function offered by the caret package was used to perform classification and cross-validation [23]. A permutation test was done to evaluate the significance of class discrimination. We built, for every permutation, a PLS-DA model between the data (X) and the permuted class labels (Y), using the optimal number of components determined by cross validation for the model based on the original class assignment. Two types of test statistics for measuring class discrimination were used. The first one is based on prediction accuracy during training. The second one is separation distance based on the ratio of the between-group sum of squares and the within-group sum of squares (B/W-ratio). Variable Importance in Projection (VIP) scores were calculated for each component. The definition of VIP score is that of a weighted sum of squares of the PLS loadings, derived from the explained Y-variation in any dimension. Also, we take into accout the weighted sum of PLS-regressions, whoseweights are defined as the functions of the sum reductions of squares through the numbers of PLS components. The average of the feature coefficients (i.e. loadings) was used to indicate the overall coefficient-based importance.

## Results

### Group composition

**Fig. 1:**
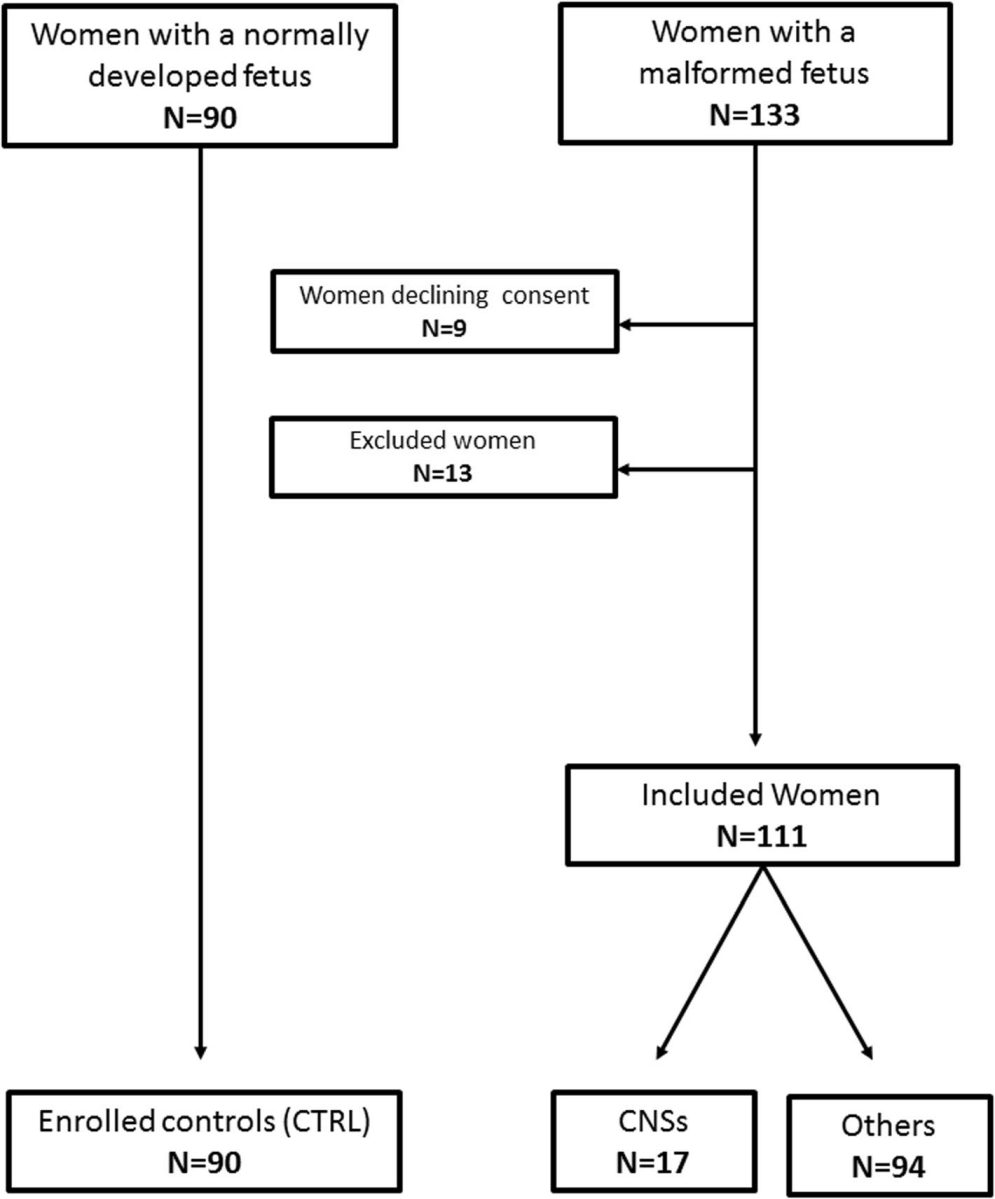
Subject selection flow-chart

One-hundred and thirty-three patients with a diagnosis of fetal anomalies were referred to University of Naples “Federico II”, University of Salerno and Hospital “G. Moscati” of Avellino, in Campania, Southern Italy, from January 2011 to December 2013.. Among these, 10 (7.5%) were excluded because they were committed to the pregnancy despite having a diagnosed fetal malformation, and 3 (2.2%) because they had a fetus with a CNS anomaly with a known genetic cause (2 fetus with Spinal Muscular Atrophy and 1 with Krabbe leukodystrophy). Among the remaining 120 patients, 111 (92.5%) gave their consent to be included in the study. Cases were compared to 90 women with a normally developed fetus (Fig. 1). Among these, 111 (55.2%) were Cases and 90 (44.8%) were CTRLs. Cases were divided into: CNS group (*n* = 17 (15.3%)) and Others (*n* = 94 (84.7%)). All the characteristics of the enrolled patients were summarized in Table 1. Distribution of fetal malformations among the study population is summarized in Table 2.

**Table 1:** Study population characteristics. HS/GED: High school/General Educational Development

Variables	Control (*n* = 90)	CNS (n = 17)	Other (n = 94)
Gestational age (weeks and days)	18w4d ± 2w1d	19w1d ± 3w1d	18w6d ± 2w1d
Maternal Age (years)	31.24 ± 6.73	31.82 ± 7.11	33.61 ± 6.31
< 30	44(48.9%)	6 (35.3%)	51 (54.3%)
≥ 30	46 (51.1%)	11 (64.7%)	43 (45.7%)
Marital Status			
Single	20 (22.2%)	2 (11.8%)	20 (21.3%)
Married	70 (77.8%)	15 (88.2%)	74 (78.7%)
Pre-preg. BMI (Kg/m^2^)			
Underweight (≤19.0)	10 (11.1%)	3 (17.6%)	13 (13.8%)
Average (19.0–24.9)	52 (57.8%)	6 (35.3%)	61 (64.9%)
Overweight (25.0–29.9)	20 (22.2%)	5 (29.4%)	15 (16.0%)
Obese (≥30.0)	8 (8.9%)	3 (17.6%)	5 (5.3%)
Education			
< high school	16 (17.8%)	2 (11.8%)	2 (2.1%)
HS/GED	53 (58.9%)	10 (58.8%)	63 (67.0%)
Any college	21 (23.3%)	5 (29.4%)	29 (30.9%)
Infant Sex			
Female	47 (52.2%)	7 (41.2%)	44 (46.8%)
Male	43 (47.8%)	10 (58.8%)	50 (53.2%)
Tobacco use			
No tobacco	80 (88.9%)	15 (88.2%)	88 (93.6%)
Tobacco use	10 (11.1%)	2 (11.8%)	6 (6.4%)

**Table 2:** Distribution of fetal malformations among the study population

Class	Sub-class	Malformations	Number of cases
Nervous system Malformations	CNS malformations	Acrania, Anencephaly, Agenesis of the corpus callosum, Hydrocephalus, Myelomeningocele, Spina bifida, Dandy Walker syndrome	17
Other Malformations	Chromosomal anomalies	Trisomy 21, Trisomy 18, Trisomy 13, Balanced translocation, Unbalanced translocation, Turner, X0/XY	44
	Multiple malformations	Multiple malformation	14
	Genetic syndrome	Major thalassemia, Cystic fibrosis, Ellis van Creveld syndrome	6
	Cardiac anomalies	Tetralogy of Fallot, Complex heart malformations	13
	Fetal Hydrops	Fetal Hydrops, Non-immune fetal Hydrops	4
	Digestive system anomalies	Budd Chiari syndrome, Bochdalek hernia	4
	Urogenital system anomalies	Kidney dysplasia, Potter syndrome	6
	Bone and skeletal system anomalies	Osteogenesis imperfecta	3

As determined during the comprehensive medical history evaluation, none of the enrolled subjects (Cases or CTRLs) were occupationally exposed to metals. The average age of the Other malformation group was 2.37 years higher than the CTRLs group (33.61 ± 6.31 vs. 31.24 ± 6.73), but the difference was not significant. The difference in age between CNS malformation and CTRLs was also not significant.

### Metal concentrations

Table 3 reports the mean serum concentrations of all metals measured among the three subject classes. The average Al concentration in the CNS malformation group (6.45 ± 15.15 μg/L) was significantly higher than both the Control group (0.11 ± .51 μg/L; *p* < 0.0006) and Other malformation group (1.44 ± 4.21 μg/L, *p* < 0.0006). Among the 90 Controls, 7 (7.8%) had a serum concentration of Al above the LOD (0.01 μg/L), while the corresponding percentage in the CNS malformation was 82.4% (14/17). An ANOVA evaluation of the Al concentration among the 8 subclasses of the “other” class showed no difference (*p* > 0.05). The average lithium concentration was higher in the Other malformation class with respect to the Control class (6.11 ± 2.17 vs. 4.73 ± 1.75 μg/L; *p* < 0.0006) whereas the average concentration of silver was lower in the CNS malformation class with respect to the Control (3.85 ± 1.39 vs. 6.19 ± 2.73 μg/L; *p* < 0.0006). No other difference was detected among the other investigated metals.

**Table 3:** Serum metal concentrations reported as natural logarithm of the concentration in μg/L. Values were reported as mean ± 1 standard deviation. * indicates significant difference (*p* < 0.0006) from the CTRL, § indicates significant difference (*p* < 0.0006) from Other malformation group

	Control (*n* = 90)	CNS (*n* = 17)	Others (*n* = 95)
Aluminum (Al)	−5.03 ± 1.27	0.14 ± 4.72^*§^	−3.79 ± 2.75
Antimony (Sb)	1.73 ± 0.88	2.03 ± 0.53	1.56 ± 1.46
Barium (Ba)	−4.95 ± 1.37	−4.19 ± 2.37	−4.73 ± 1.52
Beryllium (Be)	−1.34 ± 1.49	−2.72 ± 2.01	−2.14 ± 2.07
Cadmium (Cd)	−0.96 ± 0.89	−1.21 ± 1.38	−1.20 ± 1.51
Cerium (Ce)	−3.31 ± 0.87	− 3.43 ± 0.40	− 3.24 ± 0.66
Chromium (Cr)	0.40 ± 1.38	0.82 ± 0.56	0.82 ± 0.68
Cobalt (Co)	−0.54 ± 2.67	−1.47 ± 2.82	−0.43 ± 2.35
Copper (Cu)	7.51 ± 0.33	7.67 ± 0.37	7.60 ± 0.47
Dysprosium (Dy)	−3.33 ± 0.78	−3.93 ± 0.57	− 3.52 ± 0.93
Erbium (Er)	−3.37 ± 0.86	−4.06 ± 0.86	− 3.72 ± 1.11
Europium (Eu)	−3.51 ± 1.04	−4.61 ± 0.87	− 3.83 ± 1.10
Gadolinium (Gd)	−3.31 ± 0.81	−4.08 ± 0.93	− 3.46 ± 1.09
Gallium (Ga)	−3.36 ± 1.07	−2.79 ± 0.75	−2.90 ± 1.32
Hafnium (Hf)	−3.19 ± 1.00	−3.16 ± 0.50	− 3.03 ± 0.85
Indium (In)	−4.68 ± 0.51	−4.74 ± 0.44	− 4.57 ± 0.78
Iridium (Ir)	−3.33 ± 0.86	−3.93 ± 0.68	− 3.37 ± 0.99
Lead (Pb)	1.11 ± 1.25	1.71 ± 0.46	1.12 ± 1.88
Lithium (Li)	1.49 ± 0.37	1.55 ± 0.29	1.76 ± 0.35^*^
Manganese (Mn)	2.30 ± 0.80	2.31 ± 0.75	2.50 ± 0.72
Mercury (Hg)	1.85 ± 1.02	2.12 ± 1.22	1.40 ± 1.91
Molybdenum (Mo)	−3.09 ± 1.10	−2.48 ± 0.57	−4.12 ± 1.10
Neodymium (Nd)	−3.30 ± 0.98	−3.17 ± 0.67	− 3.32 ± 0.88
Niobium (Nb)	−3.70 ± 1.13	−4.46 ± 0.92	−4.12 ± 1.10
Osmium (Os)	−3.33 ± 0.88	−3.68 ± 1.05	− 3.31 ± 0.98
Platinum (Pt)	−3.34 ± 0.83	− 3.21 ± 0.44	−3.04 ± 0.73
Praseodynium (Pr)	−3.51 ± 0.94	−4.27 ± 1.06	−3.62 ± 1.03
Rhenium (Re)	−3.78 ± 1.09	−3.68 ± 0.98	− 3.81 ± 1.08
Rubidium (Rb)	7.12 ± 0.16	7.05 ± 0.26	7.13 ± 0.17
Ruthenium (Ru)	−3.32 ± 0.89	−3.97 ± 1.09	− 3.73 ± 1.26
Samarium (Sm)	−3.42 ± 0.94	− 3.55 ± 0.90	−3.47 ± 1.09
Selenium (Se)	5.72 ± 0.41	5.85 ± 0.35	5.59 ± 0.60
Silver (Ag)	1.70 ± 0.54	1.63 ± 0.51^*^	1.17 ± 0.52
Strontium (Sr)	2.15 ± 0.76	2.62 ± 0.46	2.24 ± 1.03
Tantalum (Ta)	−3.42 ± 0.94	−3.56 ± 0.83	−3.58 ± 0.64
Tellurium (Te)	−2.61 ± 1.13	−2.76 ± 1.80	−2.40 ± 1.53
Thallium (Tl)	−4.46 ± 1.03	−3.55 ± 1.16	−3.87 ± 1.21
Thulium (Tm)	−3.73 ± 1.04	−3.78 ± 1.27	− 3.34 ± 0.58
Titanium (Ti)	3.30 ± 0.56	3.56 ± 0.27	3.36 ± 0.64
Tungsten (W)	−3.04 ± 1.08	−3.04 ± 0.67	−2.81 ± 1.02
Vanadium (V)	−1.87 ± 2.02	−2.67 ± 2.58	−3.59 ± 1.77
Ytterbium (Yb)	−3.50 ± 0.89	−3.85 ± 1.13	− 3.40 ± 0.78
Zinc (Zn)	8.31 ± 0.52	8.62 ± 0.53	8.25 ± 1.02
Zirconium (Zr)	−0.66 ± 0.86	−0.89 ± 1.69	−1.00 ± 0.65

### Classification models

**Fig. 2:**
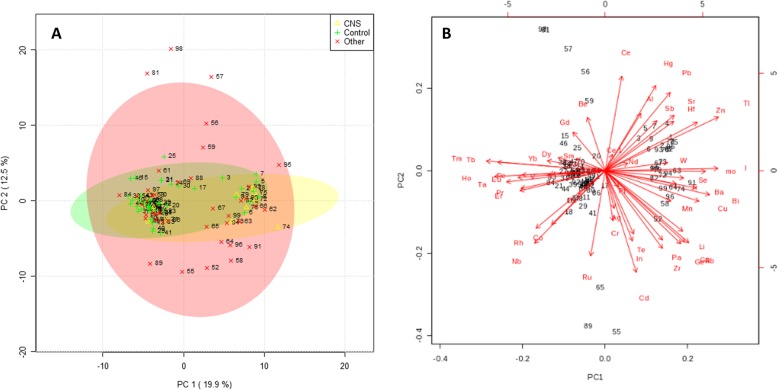
**a**. Scores plot between the first two Principal Components (PCs) in the PCA model. The explained variances are shown in parentheses. **b**. Biplot of the PCA model

**Fig. 3:**
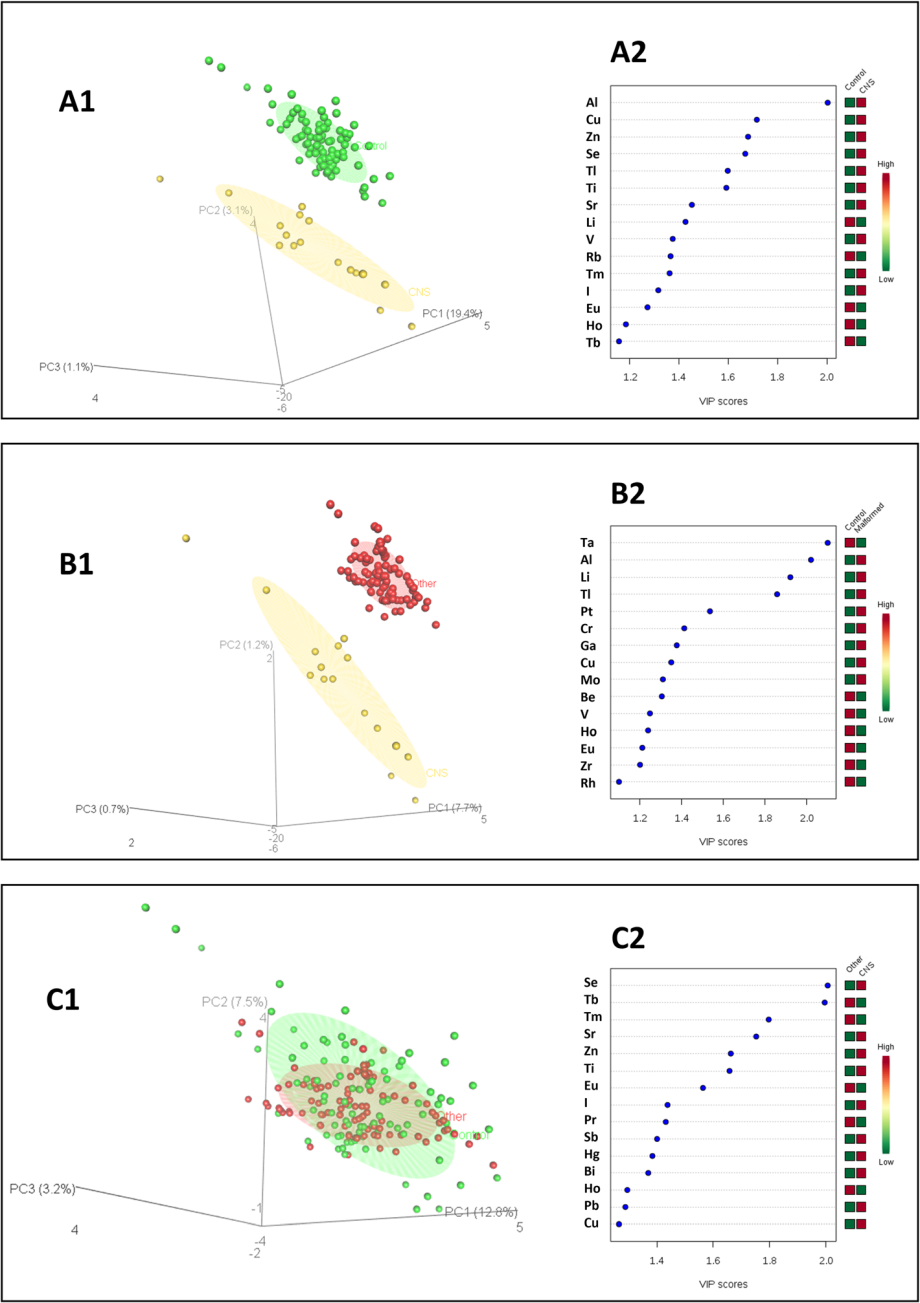
Partial Least Square (PLS-DA) model built to discriminate mothers with a CNS malformed fetus from (A) control mothers, (B) mothers with other malformed fetuses. Panel (C) shows the discrimination among the Control mothers and the mothers with other malformed fetuses

Principal component analysis (PCA) showed no natural aggregation of subjects (Fig. 2). Three PLS-DA models were built: one to discriminate CNS Cases from the Control mothers (Fig. 3a); one to discriminate all the mothers with a malformed fetus from the Controls (Fig. 3b); and one to separate the mothers with a CNS malformed fetus from those with other malformations (Fig. 3c). Variables most responsible for class separation were identified in each PLS-DA model. For the first model (CNS Vs Controls), Al showed the higher VIP score (VIP score > 2). For the second model (All malformations Vs Controls) both Al and tantalum showed a VIP score > 2, while in the third model, both selenium and terbium showed a VIP score > 2.

## Discussion

Our results show that there is an association between serum Al concentration and CNS congenital defects. In particular, serum Al was significantly higher in mothers with a fetus affected by a CNS congenital defects whereas Al was not even detectable in the great majority of serum samples coming from mothers with a normally-developed fetus (Table 3). Moreover, multivariate analysis confirmed that the Al concentration is the variable that is most responsible for class separation (higher VIP-score). In the multivariate model discriminating all malformations vs. controls, both thallium and Al showed VIP scores > 2. Thallium has already been reported as teratogenic for rats [24]. However, our results showed no difference in Thallium concentration among the studied groups (see Table 3). No difference was found even after further subdividing the Others class. This can be due to the low sample size or to the very low reported Thallium concentration. Al is ubiquitous on Earth and humans are exposed to it through different routes: absorption through the gastrointestinal tract, the skin, and the epithelium of the upper and lower respiratory tract [25]. It is present in water and food such as tea and juices, salt, spices, processed cheese, infant formula, fish and shellfish [26]. It is also present in antacid drugs, antiperspirants and airborne particles [27] [28]. Al is able to reach and accumulate in the human brain [29]. Chronic exposure to Al has been associated with neurodegenerative diseases, such as Alzheimer Disease [30], while its acute neurotoxicity has been directly implicated in the onset of dialysis encephalopathy [31] [32].

Al developmental toxicity on animal models has been extensively reviewed by Domingo [33], showing a certain degree of embryo-fetal toxicity and teratogenic effects, as well as decreased fetal weight, in pregnant mice and rats treated with Al at different doses, in different chemical forms and following different routes of administration. There is also evidence linking Al exposure and the prevalence of NTDs in humans [13] [14] [15]. Data from an American study show that normal serum levels of Al in healthy people are approximately 1–3 μg/L [34], while according to the Italian Reference Values Society (SIVR), serum Al concentration in healthy people ranges from 1 to 6 μg/L [35]. The available evidence suggests that maternal Al is able to cross the placental barrier and reach fetal circulation. In a study by Kruger et al. [36], examining human placentas at term, Al was detectable in almost all placental tissues and membranes analyzed, while it was detectable in only half of the analyzed umbilical cords. Mikelson et al. [37] also reported detectable concentrations of Al in term placental tissue. These results suggest that placental tissue is able to act as a filter for Al passage, but that this filter is saturable. The observed placental accumulation of Al may help explain the difference between the normal serum Al range in the non-pregnant population and the values reported in our study [34] [35]. Yumoto et al. [38] found that the brain of rat fetuses incorporated ^26^Al subcutaneously injected to their mothers starting from day 21 of gestation. These results may not be generalized to the first weeks of pregnancy that are important for brain development. However, Anane et al. [39] report Al accumulation in amniotic fluid and fetal organs (including brain) after transcutaneous Al administration in pregnant rats for 20 days starting from the onset of gestation. Alternatively, studies reviewed by Borak and Wise [40] did not show increased Al accumulation in rats exposed in utero.

As mentioned, Al is able to reach the brain, either through the olfactory route [29] or crossing the Bblood-brain barrier (BBB). Because Al circulates in blood predominantly as Al-Transferrin, it reaches the CNS through transferrin receptor-mediated endocytosis at the BBB capillaries [27] [41]. A crucial aspect regarding Al toxicity (and neurotoxicity in this case) is that, since Al is devoid of biological significance in the human body, any bioavailable amount is potentially toxic [29]. The molecular mechanisms underlying Al neurotoxicity are extensively reviewed by Verstraeten et al. [42]*.* Although it is not a redox metal, Al can cause oxidative stress by enhancing Fe-mediated oxidative damage [32] [42]. Membrane phospholipids are particularly susceptible to this damage, especially those containing unsaturated fatty acyl chains. Myelin is another preferential target of Al-mediated oxidative damage. It has been demonstrated that prenatal Al exposure in mice increases myelingalactolipid content. As a consequence, segregation of clusters of phospholipids in which oxidative damage may progress could be promoted. Al exposure may also cause loss of cellular membrane fluidity (i.e. increased membrane rigidity) [43] [44] [45]. Al is also regarded as being excitotoxic, since it elevates intracellular calcium, with consequent increased molecule phosphorylation [25] [46]. Al exposure can also inhibit Phosphatidylinositol 4,5-bisphosphate (PIP2) hydrolysis by Phospholipase C (PLC) in prokaryotic cells [47]. In human neuronal cells, PLC-mediated pathways play an important role not only in neurotransmission, but also during brain development [48] (see Introduction). In Neuro-2a cells, Al-maltolate treatment caused increased p53 and BAX expression and decreased Bcl-2 expression, leading to cell apoptosis [49]. A contribution to understanding in vivo Al-induced oxidative stress comes from Sharma and Mishra [50]: the contemporary administration of Al and antioxidants, such as Tiron and/or Glutathione, in pregnant rats significantly reduced fetotoxicity compared to pregnant rats exposed to Al only. All the described mechanisms of toxicity can be related both to neurodegeneration and to altered brain development. However, a pathogenetic relationship between the mechanisms of Al toxicity and the onset of congenital defects of the central nervous system is far from being completely understood. Overall, our work provides further evidence of embryo/fetotoxic effects related to exposure to this metal. Strengths of our study are related to the homogeneity of the included population in terms of gestational age at samples’ collection and exposure to the same environment; this is particularly advantageous in order to minimize biases. Moreover, we investigated a metallome, determining the concentrations of multiple metals present in the serum: thus, the significant difference found in serum Al concentration between CNS Cases and Controls stems from the investigation of the entire metal burden. However, a possible pathogenic interference played by this metal burden should not be excluded and needs further study.

The elevated Lithium levels founded in the Other class are coherent with the suspect of association between lithium and cardiac malformation reported by Patorno et al. [51] [52].. However, there is not sufficient evidence to justify an association between elevated lithium level and cardiac malformations.

On contrary the low level of silver in the CNS malformed fetuses was not yet reported, We found only a study reporting that silver nano-particles can reach the fetus trough the placenta transfer [53] and can cause skeletal dysmorphology in rats. Anyhow, it can not justify our opposite finding of lower silver level in CNS fetuses.

The small number of CNS samples is the major weakness of our study. In Europe, fetal anomalies represent a rare pregnancy-related event. The European Surveillance of Congenital Anomalies (EUROCAT) reports a global occurrence of fetal anomalies in 2.39% of all pregnancies [1]. Only 17.6% (0.42% of the total pregnancies) of fetal anomalies-complicated pregnancies are aborted and only 0.04% are related to a CNS anomaly. According to this data, it is difficult to obtain an adequate number of CNS Cases that are able to be sampled. Thus, our relatively few CNS Cases, is more appropriate than would initially seem. Other peer-review studies in this field were conducted using far fewer cases [54]. However, the small sample size did not give us the possibility to assess if there is a difference in Al levels among different types of CNS malformations. This topic could be further discussed in future studies with a larger sample size.

Moreover, we decided to exclude cases that continued the pregnancy regardless of the diagnosis of a fetal malformation. This is because the majority of them decided to deliver in other hospitals, making it difficult to follow up with them. We can not exclude that this choice could contribute to a selection bias, however small.

## Conclusions

Aluminum may play a role in the onset of central nervous system malformations, although the exact Aluminum species and related specific type of malformation needs further elucidation.

While sur data provide valuable insight into one of the multifactorial causes of CNS anomalies, further investigation of specific Al-mediated teratogenic mechanisms is neededs.

## Data Availability

All the data used for this paper are available on request from corresponding author.

## Abbreviations

Al: Aluminum
C PLC: Phospholipase
CNS: Central Nervous System
CTRLs: Controls
EUROCAT: European Surveillance of Congenital Anomalies
ICP–MS: Inductively Coupled Plasma Mass Spectrometry
LOD: Limit of detection
Mn: Manganese
NTDs: Neural Tube Defects
PCA: Principal Component Analysis
PIP2: Phosphatidylinositol 4,5-bisphosphate
PLS-DA: Partial Least Squares-Discriminant Analysis
VIP: Variable Importance in Projection
